# The odd couple: using biomedical and intersectional approaches to address health inequities

**DOI:** 10.1080/16549716.2017.1326686

**Published:** 2017-06-22

**Authors:** Olena Hankivsky, Lesley Doyal, Gillian Einstein, Ursula Kelly, Janet Shim, Lynn Weber, Robin Repta

**Affiliations:** ^a^ School of Public Policy, Simon Fraser University, Vancouver, BC, Canada; ^b^ Health and Social Care, School for Policy Studies, University of Bristol, Bristol, UK; ^c^ Department of Psychology, University of Toronto, Toronto, ON, Canada; ^d^ Atlanta VA Medical Center, Emory University Nell Hodgson Woodruff School of Nursing, Atlanta, GA, USA; ^e^ School of Nursing, University of California, San Francisco, CA, USA; ^f^ Department of Psychology, University of South Carolina, Columbia, SC, USA; ^g^ Interdisciplinary Studies Graduate Program, University of British Columbia, Vancouver, BC, Canada

**Keywords:** Intersectionality, biomedicine, health inequity, interdisciplinary

## Abstract

**Background**: Better understanding and addressing health inequities is a growing global priority.

**Objective**: In this paper, we contribute to the literature examining complex relationships between biological and social dimensions in the field of health inequalities. Specifically, we explore the potential of intersectionality to advance current approaches to socio-biological entwinements.

**Design**: We provide a brief overview of current approaches to combining both biological and social factors in a single study, and then investigate the contributions of an intersectional framework to such work.

**Results**: We offer a number of concrete examples of how intersectionality has been used empirically to bring both biological and social factors together in the areas of HIV, post-traumatic stress disorder, female genital circumcision/mutilation/cutting, and cardiovascular disease.

**Conclusion**: We argue that an intersectional approach can further research that integrates biological and social aspects of human lives and human health and ultimately generate better and more precise evidence for effective policies and practices aimed at tackling health inequities.

## Background

In recent years health inequities have become a growing concern among many international organizations [1–8]. Recent efforts, including the United Nations’ (UN’s) Sustainable Development Goals, have focused on concrete actions to reduce these inequities [9–11]. At the same time the World Health Organization (WHO) has also stressed the importance of theoretical and methodological innovations in health research, to which this paper is a response [12].

We investigate the integration of two approaches often thought to be in opposition – biology/biomedicine on the one hand and an intersectional approach to social science on the other. There are fundamental tensions between the two because of differences in perspectives and methodologies. Put simply, biomedicine has usually taken a reductionist approach focused on the physiology of the body, with health and illness understood and treated as internal to its various parts [13]. Conversely social scientists have tended to overlook biological aspects of human experience in favor of focusing on the social, economic, and historical contexts shaping health and illness [13–16].

In light of these different epistemologies, some have argued that researchers from biomedical backgrounds may find using intersectionality daunting [17]. However, the potential synergies between biomedicine and social science have increasingly been recognized, and interdisciplinary collaboration has become more common. Nevertheless, more attention is needed to develop the full potential of this approach for transforming knowledge production related to health, illness, and well-being [16,18–23]. In particular, such work will need to focus much more on weaving together biology/biomedicine with a social science approach that is explicitly intersectional.

To contextualize our contribution, we begin this paper with a brief overview of two current approaches to the integration of the biological and the social. The first refers to a number of attempts to integrate sex and gender while the second involves what has been called the ‘ecosocial model’ [24–28]. We then explore how an intersectionality framework can extend and improve both of these moves towards integration. To demonstrate the value of an intersectional approach, we provide concrete examples in the areas of: HIV, post-traumatic stress disorder (PTSD), female genital circumcision/mutilation/cutting (FGC), and cardiovascular disease (CVD). Finally, we argue that an intersectional approach of this kind can provide better evidence for more effective policies and practices aimed at the tackling of health inequities.

### Biomedical approaches and their evolution to biological approaches

Biomedical approaches have played a central role in health research, providing the basis for more rigorous clinical knowledge, effective interventions, and predictive models of disease and illness. The positive outcomes have included prevention of and/or cures for communicable diseases (e.g. vaccination for polio, smallpox, and diphtheria); public health measures for cholera and typhoid; effective treatment of non-communicable diseases (e.g. chemo- and immunotherapies, pharmaceuticals, and surgeries); safe child birth practices; assisted reproductive technologies; and, more recently, discoveries with important therapeutic potential such as the isolation of stem cells, and the mapping of the human genome.

These latest advances have led to the development of what is often referred to as ‘personalized’ or ‘individualized’ medicine, in which an individual’s genomic imprint is used to predict one’s susceptibility to disease and also to tailor individual treatment [29]. This new approach warrants a shift in language from biomedical to biological approaches to health research. However, it does not yet incorporate *social characteristics* such as gender, socio-economic status (SES), education, and ethnicity – all of which have been shown to powerfully influence and shape biology, including disease and illness outcomes.

In the absence of reference to these social dimensions, the limitations of the personalized medicine paradigm are apparent. First, the model gives primacy to biological explanations of health outcomes, focusing on the body as an island unto itself, and defining illness as primarily internal. Research is limited to exploring deterministic factors at the level of the individual body [13,30,31]. Explanations of variations in health are reduced to constructs such as sex and race that are taken to represent biologically innate characteristics and are measured as independent and discrete variables [32]. Rates and distributions of diseases and illnesses are then seen to result from individual-level characteristics, which can be aggregated to broad generalizations about population health [33].

Moreover, when variables such as gender, race, and class are incorporated, they are ascribed only a biomedical relevance, that is, they are equated with biological disease risk and/or the lifestyles and behaviors that place a person ‘at risk’ for a disease or medical condition [34–36]. The effect of these intersecting social locations on the biology of the individual body has not been adequately investigated and, as a consequence, important avenues for understanding poor health and health inequities remain unexplored.

Yet, alongside (but not integrated with) the development of personalized medicine there has been a growing understanding and acceptance of the social determinants of health (SDH). The fact that health is determined by far more than biology or indeed health care systems is now widely accepted [7,10,11,37,38]. Hence, it is necessary to ensure that biological approaches contribute, in tandem with social perspectives, to achieve more complete understandings of health.

### Socio-biological entwinements

To date, a number of important conceptual advancements have captured the complex and dynamic interplay of biological and social dimensions, demonstrating how ‘social inequalities become embedded in our biology’[39,p.3]. Work on the integration of sex/gender provides one noteworthy example. Scholarship in this vein includes Fausto-Sterling’s dynamic systems theory [40–42], Bekker’s Multi-Facet Gender and Health Model [43], Bird and Rieker’s ‘constrained choices’ multi-level model [44], and Annandale’s ‘new single system’ [45]. Guidelines for applying these concepts to health research have also been developed [17,46]. Springer, Stellman, and Jordan-Young [47] have developed good practice guidelines for research on sex, gender, and male–female health differences, while Ritz et al. [48] have proposed an approach for basic experimental researchers to take sex and gender differences into account.

A key argument for integrating sex/gender into health research is to redress the historic exclusion of women and female animals from most studies [49–51]. The inclusion of women and female animals is important for the promotion of gender equity [52–57], as well as being necessary for the promotion of scientifically rigorous and relevant research findings [58–61]. Increasingly, the limitations of prioritizing sex/gender have been noted as this can exclude other factors, thereby undermining the complexities of health experiences and outcomes [14,62]. Even those advocating for sex/gender analyses are now asking: ‘How do we measure diversity and its interaction with sex and gender?’ and more specifically, ‘What influence do intersectional-type analyses have on the way sex and gender are integrated into health research?’[53,p.12].

Arguably the most comprehensive and leading-edge approach to integrating biological and social approaches is Nancy Krieger’s ecosocial model [24]. Developed in the context of epidemiology, its central focus is on the ways in which humans ‘embody’ their social and economic contexts and how these result in a variety of inequities in patterns of illness and disease. Analytic attention is paid to the ways in which health is shaped over the life course by different forms of social inequality operating at multiple levels. The approach also highlights differences between groups within a standard population category, within-group differences, and how agency and resistance mitigate the lived experiences of social inequality.

Recently researchers have argued that the addition of intersectionality to the ecosocial approach can enhance its explanatory capacity further. As Agenore et al. argue, ‘intersectionality provides empirical researchers with a theoretical basis for conceptually and operationally identifying how multiple dimensions of social inequality simultaneously influence population health, including health inequities’[63,p.111]. The authors attempt to illustrate their argument by using both ecosocial and intersectional models to investigate how sexual orientation, sex of sexual partners, and race/ethnicity jointly influence Pap test use among black, Latina, and white US women [63]. However, they do not explicitly discuss why both approaches were needed, what makes them complementary, and perhaps even more importantly how they are distinct. These issues are therefore explored in more detail in this paper in order to show how specific aspects of intersectionality can extend Krieger’s important work on the explication of socio-biological entwinements in health.

## Intersectionality

As a term, ‘intersectionality’ was coined by American critical legal race scholar Kimberle Williams Crenshaw [64], but the central ideas of intersectionality have deep historic roots within and beyond the US. Black activists and feminists, as well as Latina, queer, post-colonial, and Indigenous scholars have all sought to articulate the complex factors and processes that shape human lives [65–68]. Intersectionality is a promising resource to advance health inequities research [69–72]. While it has been defined and utilized in various ways, for the purposes of this discussion we refer to it as a framework which focuses on the ways in which multiple axes of social inequality intersect and co-construct one another at the macro and micro levels to produce a broad range of unequal outcomes, in both individual and population health [15,73–77]. These interactions occur within a context of connected systems and structures of power (e.g. laws, policies, state governments and other political and economic unions, religious institutions, media). Through such processes, interdependent forms of privilege and oppression are shaped by colonialism, imperialism, racism, homophobia, ableism, and patriarchy[78,p.2].

When compared to Krieger’s model, there are a number of defining features of intersectionality that can be drawn on to extend the ecosocial approach. Specifically, intersectionality prioritizes interactions and complex relationships between social locations and systems of power while emphasizing the *simultaneity* of privilege/penalty, which is so often ignored in health inequities research. It also privileges diverse sources and forms of knowledge beyond those typically found in social epidemiology (the foundational grounding for the ecosocial model) including, for example, lay knowledge from the point of view of affected/subordinated groups as a point of departure [79], and places importance on mixed methods for the production of evidence. Intersectionality requires self-reflexivity by researchers and policy actors to ensure that those who shape the production of evidence and influence political decisions are aware of their power, values, and position and how these affect the kinds of research questions that are asked, how research is conducted, and how research evidence is used and implemented. And finally, intersectionality-informed research transcends the mere description of health inequities and focuses on the goal of social justice as a mechanism for social change and transformation.

Although intersectionality’s promise in the context of public health is now well established, critics have also pointed out its marginal attention to the biological and have noted this as an area requiring far more reflection [14–16,23]. At a conceptual level, a number of scholars [21,80] have proposed the use of the state of the body itself as an additional category to be treated in the same way as others such as SES, ethnicity, or gender. They argue that this would avoid privileging biology through biological reductionism but so far there has been little effort to address this gap either conceptually or empirically.

## Combining biological approaches and intersectionality

Integrating intersectionality and biological perspectives requires well-considered decisions at every stage of the research process, from conceptualization of the problem and study design and implementation to interpretation of findings [16]. This involves establishing intersectoral and transdisciplinary collaborations among partners and researchers committed to communicate and work across systemic power-based inequities in the research enterprise. Another key aspect of integrating intersectionality and biological approaches is seeking a balance between methods and meaning [23]. For example, the biomedical emphasis on measurement and quantification can impede the elimination of hierarchies of health; and the largely qualitative methods commonly used in intersectionality-guided research often reveal the meaning of inequities while failing to assess the effectiveness of different health initiatives and interventions.

The very diverse research examples that follow, which range from explorations of broad global pandemics to more detailed stand-alone research studies, represent some of the few attempts to date to draw on an intersectional framework to advance understandings of socio-biological entwinements. Despite their marked differences, together our secondary analysis of their content shows the transformative effects of intersectionality. Specifically, we used key elements of intersectionality to analyze and unite the examples. Accordingly, each study demonstrates how it:

- addresses multiple systems of inequity simultaneously;
- utilizes multiple levels of analysis, including the biological, interpersonal, institutional, and societal;
- situates research in time and place;
- engages in research methods that privilege the perspective of multiple subordinated groups; and
- prioritizes a commitment to social justice.

We begin with a structural overview of the intersectional dimensions of the global HIV pandemic and follow with more specific case studies of PTSD, FGC, and CVD.

### Example #1: using an intersectional approach to explore diversity and inequity in the global HIV pandemic

The burden of HIV offers a valuable case study for drawing together the intersections between the biological, the economic, the social, and the cultural elements of what has come to be seen as the modern plague of the twenty-first century. The disease was first identified in the US in the 1980s among men who had sex with men (MSM). As the relationship between ‘gay’ sexual identities and practices and HIV gradually became clear, those affected were increasingly stigmatized [81]. Thus, existing heterosexist cultures played a significant part in shaping negative attitudes towards those faced with a specific biological condition. The risks of this ‘lifestyle’ were also shown to be exacerbated by the fact that anal sex is the most dangerous form of intercourse from a physiological point of view [82]. As a result, gay men (and women), as well as other activists, joined together to fight for respect and for resources in what was the most dramatic politicization of an illness in modern times.

At the same time, the incidence of HIV and AIDS began to move beyond this initial group. In the US it spread well beyond the community of MSM to include residents of inner cities, many of whom were black or Latina/o/x and already experiencing poverty and racism. Sex workers and injection drug users were especially common among this group, creating additional discrimination against already marginalized groups. It was of major significance that this expanding HIV-positive population included both women and men. Thus, both biological sex and social gender became increasingly important variables in attempts to map and to explain the nature of the disease. The concept of a ‘gay plague’ with the main focus on sexuality could no longer be deemed to be of either scientific or moral value. Hence, the shift towards what can be seen as an intersectional analysis became increasingly important.

By the year 2000, some 75% of all those who were HIV-positive were in the African region with the spread rapidly following into Asia and also Eastern Europe in particular. Hence, it was clear that the world was not faced with one homogeneous pandemic but diverse epidemics of the same disease in different settings, spread by a range of means among varied populations. It is in this context that we can most easily identify the particular value of an intersectional approach [83].

As the virus spread outwards to other groups, a broader range of determinants came into play. Most importantly both sex and gender took on greater importance in both science and policy making as heterosexual intercourse became the dominant mode of transmission, with women now making up about 50% of HIV-positive people worldwide and more than 60% in the African region [84]. From a biological perspective, women are more vulnerable than men to infection from a single encounter. The act of unprotected heterosex results in potentially infected semen remaining in contact with vulnerable vaginal tissues for what may be lengthy periods. This risk can be exacerbated by the fact that many women (especially the poorest) have both untreated gynaecological illnesses as well as traumatic injuries that make vaginal tissues more vulnerable [85]. Hence in this context the material process of biological transmission of the HIV virus between women and men must be incorporated as part of an intersectional approach to the disease.

But these biological differences cannot be seen as the only drivers of what has been called the ‘feminization’ of the pandemic. There is now an extensive literature linking HIV with wider gender divisions in society [86]. The most obvious connection is the male domination so frequently experienced in heterosexual encounters. This can be enacted in a number of different ways.

Most importantly women may be unable to prevent men from forcing them to have unprotected sexual activity either because they are threatened with violence and/or because they are linked to men through legal ties and/or economic dependence. In many parts of the world wives are expected to have sex at their husband’s behest, while those in more informal relationships may be afraid of losing financial support for themselves and their families if they do not respond to the demands of their partners. It is important to note that gender may put men at risk too. This reflects cultural rather than economic influences, as many men may feel pressured to ‘prove’ their masculinity through frequent and often unprotected sex [87].

Thus, there are clear intersections between sexuality, biological sex and social gender, and patterns of income and wealth in shaping the variety of HIV epidemics. However, these interconnections are not as straightforward as is often assumed. On the one hand, poverty played little part in the case of men infected through sex with other men in the early stages of the pandemic and this remains the case in most parts of the world. But as heterosexual practices have become major sources of infection in the less developed parts of the world, intimate relationships between individuals have increasingly been shaped by the economic rather than the emotional needs of those involved.

To make matters even more complex, the impact of income and wealth on HIV infection cannot be read from economic status in any straightforward way. Surprisingly perhaps, in Africa, those (usually urban dwellers) who have access to the highest incomes are more likely to become infected than their low-income compatriots. This has generated considerable debate with the most common explanation being that wealthier men are likely to be able to afford more relationships with (usually younger) women and hence to put themselves at greater risk [87,88].

Viewed from a population level, it is the poorest countries that have the largest absolute numbers of people with HIV infection. And it is here that incorporating ‘geopolitical status’ into an intersectional model is especially important. Those many millions who live in deprived settings are likely to have been both physically and psychologically weakened by their circumstances, and hence are more vulnerable to a wide range of infectious diseases. Lack of basic infrastructure such as water and sanitation as well as inadequate nutrition will all contribute to the failure to meet the basic human needs required for positive health. Similarly lack of basic medical care across the life span will not only enhance vulnerability but make life much more difficult and probably shorter for those already infected. Hence, the increasing inequalities built into the world geopolitical system provide a basic foundation for making sense of the past and future of the pandemic.

This brief account has shown that the 35 million people living with HIV are by no means a homogeneous group. Though they may all be attempting to survive the same disease, they will have very different levels of resources at their disposal. Variations in the settings in which they live, their status in their community, and the nature of their intimate relationships will generate inequalities in their capacity to meet their basic human needs, to access health services, and to preserve their autonomy and their sense of their own identity. The use of an intersectional analysis can be of vital importance in identifying the ways in which these complex processes continue to shape the global epidemics, to limit their further spread, and to develop more equitable and effective care for those already infected.

### Example #2: PTSD – elucidating the role of power differentials on outcomes

The second example moves from the global to the local to illustrate how understandings of PTSD can be fundamentally transformed by an integration of biomedical and intersectional perspectives [16,89]. A community-based, mixed methods study with Latina women who experienced intimate partner violence (IPV) sought to develop (1) acceptable and effective treatment for PTSD; (2) local community-based, accessible mental health treatment resources; (3) clinical and research collaborations with community partners, and to address unequal power relationships between the community (activists, service providers, and residents) and the health care system in the community. As detailed next, the inclusion of intersectionality transformed each step of the research process, generating insights that transcend biomedical/biological or ecosocial approaches.

#### Conceptualization of the problem

A biomedical rationale for developing scientifically grounded treatments for violence-related PTSD in general would include a focus on morbidity, mortality, and social and economic costs associated with PTSD. PTSD appears to be the link between exposure to violence and poor health outcomes, as well as social and occupational hazards, role functioning, and risky behavior [90]. The ecosocial approach to the problem of PTSD treatment for immigrant Latino women who experienced IPV would take into consideration multiple social and economic factors. For example, the political climate in the US in the past decade has vilified the immigrant population, particularly those who are undocumented. Federal agencies and local police forces have created a climate of fear among some immigrant populations via aggressive identification and deportation of undocumented immigrants. National and state entitlement programs, such as unemployment benefits, job training programs, public education, and Medicaid, are not available to this population. All of these social and economic factors create an increased health burden within this population, increasing the likelihood that they will have PTSD and that they will not have access to treatment for PTSD.

Conversely, an intersectional approach frames the problem as one of power inequities at multiple levels – interpersonal, institutional, and societal and multiple systems (race, ethnicity, gender/class). For example, the women in the study are Latina, immigrant, undocumented, and victims/survivors of IPV. Many have limited English proficiency, low income, mental health problems, and limited access to health care. They typically lack a social safety net by virtue of an absent supportive familial and social network and their attendant social isolation. Each of these marginalized positions interacts with the others and results in ‘intersectional invisibility’ [91], where experiences of people with intersectional subordinate group identities are misrepresented, marginalized, and disempowered. Every woman in Kelly’s study had a unique experience of identity, disadvantage, and inequality, creating individual-specific multiple jeopardy [92] and universal social injustice. The integration of biomedical and intersectional approaches in this study meant that both the women’s PTSD systems and intersectional invisibility were acknowledged and addressed throughout the research study.

#### Research approach

This study, conducted purely within a biomedical model, could have been designed, initiated, and conducted by the researcher without involving the affected community and other stakeholders. The study objective would be to evaluate the effectiveness of the new intervention in reducing PTSD symptom severity, the outcome measure of the study, in this defined population. Analyses might include the degree of statistical association between IPV type (physical, sexual, and possibly emotional abuse) and PTSD symptom severity or responsiveness to the intervention, since IPV is the identified trauma causing PTSD in this study. The study design and procedures would remain unaltered in order to maintain scientific rigor, a requisite for the study findings to be considered reliable and valid. The study would be conducted in the controlled environment of a health care setting or research lab to minimize the introduction of confounding influences as well as for researcher convenience and comfort. Inclusion and exclusion criteria would be designed to establish the most homogeneous sample possible, reducing confounding influences on the results.

In the ecosocial model, these confounding influences, i.e. external factors, including, among others, social, economic, cultural, and historical context, would be defined and incorporated into the study as individual, independent, and quantifiable influences on the women’s health and on the effectiveness of the intervention. These would be examined from an epidemiologic angle – which of these influences leads to health disparities and to what extent?

An alternative, suited to the integration of intersectionality and biomedicine, is community-based participatory research (CBPR) – a research approach that engages community partners and researchers as equal collaborators who mutually participate in a research endeavor [93]. In this study, the CBPR approach resulted in several research processes and decisions that would not be present in either biomedical or ecosocial model-driven research [89]. A few examples include the community partner involvement in establishing the need for the study, the fact that the setting was a community-based agency, the intervention was informed by agency staff and participants, and that focus groups and individual data were collected throughout the study, enabling formative evaluation and study revision as indicated.

#### Multidimensional operationalization and measurement of ‘discrete’ variables

In biomedical research, dimensions of social inequity are typically conceptualized as demographic variables and measured at the nominal level by mutually exclusive categories. From an intersectional perspective, this is particularly problematic when it comes to inequities centered in power relations such as immigrant, culture, and Latino. In this study, these constructs were measured in multidimensional ways whenever possible, while at the same time recognizing that this approach fails to completely capture the essence of a person’s social identity, location, and experience.

In biomedical research, ‘immigrant’ is typically treated as a unidimensional variable, a single measurement that fails to capture the aggregate dimensions of the construct ‘immigrant.’ In this study, integrating an intersectional approach led to the decision to use multiple measures of ‘immigrant’: legal status, years living in the US, acculturation, and English proficiency. While this list was not comprehensive, it captured multiple experiences of ‘immigrant-ness,’ beyond the literal definition of an immigrant as a person who comes to live permanently in a foreign country. For the women in this study, their immigration status, legal or illegal, intersected with their IPV (e.g. the experience of fear in calling the police to intervene and risking deportation, their degree of English proficiency influenced their ability to seek support or access health care and social services).

Intersectionality also guided the interpretation of ‘culture’ in this study. In the biomedical approach, culture generally refers to ethnicity, in this case, Latino. Though it was recognized at the outset that there is not one ‘Latino’ culture, there were few studies of PTSD interventions that had been tailored for any ethnic group. Qualitative data related to Latino culture were collected at the beginning of the study to guide the design of the intervention. However, qualitative data collected post-intervention indicated that, for the women in the study, culture had more to do with experiencing IPV than ethnicity. ‘Culture’ for them meant shared experiences of lack of power in their intimate relationships and the intersection of that powerlessness with their other social locations. A recurring comment was, ‘We are all the same because we experienced the same thing. It doesn’t matter where you were born or where you are from.’

While they recognized their ‘sameness’ as women who experienced IPV, the women (and the staff and researchers) also recognized the multiple, variable, and ever-changing influences on their lives that made their experiences, degree of power, and options and opportunities unique, which, as mentioned previously, created individual-specific multiple jeopardy [92] and universal social injustice. The shared responsibility of the community partner and researcher was to address both of these, through developing and adapting the intervention, as well as providing additional resources to the women, and advocating for changes in health policy and legal systems that were harming the women beyond their overt oppression through IPV.

### Example #3: FGC – the entwinements of traditional practices, biology, gender, and race

FGC is a traditional practice carried out in many regions of Northern Africa on the bodies of girls from infancy to young adulthood (depending on the region). To a large extent, from the perspective of many who study FGC and produce health guidelines, as well as laws criminalizing the practice in the West, women with FGC are considered disempowered #x2013; regardless of their social standing or class – and mutilated in body regions involved with reproduction and sexual pleasure. It is important to note that the Somali women we studied did not consider themselves disempowered. When tackling the ethics committee’s concern that they would be at risk of angering their husbands by participating in our study, they said, ‘You tell them nobody tells Somali women what to do’ [18]. Designing a study of its long-term repercussions, especially with respect to its effect on the central nervous system, however, requires consideration of the entwinements of biology with gender and race within the context of multiple systems of inequality and across the life course.

#### Methods of studying FGC

When approached from a purely biomedical view, the focus is most often on the reproductive health and genitalia of the women; such studies highlight difficulties in labour, urine and blood retention, obtaining sexual pleasure, and the immediate effects of the cutting [94]. It is worth noting that focus on the genitals has obscured understanding of other real health issues such as CVD, even when there are reports of pre and co-morbid conditions [96]. What is needed is an approach that simultaneously takes into account biology and meaning from the perspective of gender, race, and immigration, privileging the perspective of differently situated women with FGC. An intersectional approach allows for the consideration of these factors in the design, execution, and interpretations of data, facilitating the interactive merging of biological and social dimensions within a multilevel research project.

#### Understanding chronic pain in women with FGC

A recent study led by the neuroscientist Gillian Einstein [18] used an intersectional approach showing how bodily effects of FGC affected not only the reproductive system but the wider nervous system through the cutting of nerve and muscle with the consequence of neural rewiring. FGC was thus seen as leading to nervous system changes (central and peripheral) [18,94], which led to different gendered behaviors, sensations, and experiences of being in the social world. Since one index of neural rewiring is chronic pain, the study investigated whether or not a sample of Somali Canadian women in the Greater Toronto Area experienced such pain [94]. A community-based study was set up that asked about chronic pain both from the perspective of the women in the study and from the observations of their bodies’ reactions to physiological testing. Researchers mixed methods that are often seen as oppositional, using qualitative, quantitative, and physiological measures, labelling such a combination ‘Very Mixed Methods’ (VMM) [18]. This ensured socio-biological entwinements allowing for comparisons across the information about how the women themselves felt in the context of their FGC, and their physiological response to touch.

Moreover, the methodological approach broke down hierarchies on many levels. First, the hierarchy between researcher and participant was leveled by engaging with a Community Advisory Group, who gave input on everything from the usefulness of the questions to the instruments used. Women’s stories were privileged. Hierarchies of body systems were leveled by subordinating the nervous system to what happens at the site of the reproductive system; we challenged the view of women with FGC as being only about reproductive health by being interested in their brains and not just their genitalia [18].

#### Interpretation of findings

Findings about pain were interpreted in the context of Somali meanings of pain, revealing that while Somali-Canadian women with FGC had what biomedicine would label a ‘chronic, neuropathic pain condition’, the women studied considered this to be a normal part of being a woman. An intersectional approach showed that the category of ‘Somali, woman, immigrant’ could be further divided between those who immigrated from the city or the country, those who grew up with economic privilege and those without, and those who were given a local anesthetic prior to their circumcision and those who were not [18]. The intersections of these social aspects influenced their interpretation of their pain. Women who were anesthetized during the procedure described their recovery experience – the weeks in bed, the pain on peeing, and the preferential treatment they received such as being fed before the boys and men, being given meat and milk to eat. Women who were not anesthetized focused on the surprise, pain, and feelings during the procedure.

The authors call the combination of these approaches, along with reflexivity about the project, ‘situated neuroscience’ [18]. This involves an intersectional view on the nervous system allowing consideration of multiple levels of analysis, privileging the perspective of the group, situating the research in time and place, and treating the brain as part of the rest of the body and not a privileged bodily location. Using intersectionality to create a situated neuroscience also begins to allow a view of the body as in constant communication with the social world, each affecting and interacting with the other or, as Grosz describes it, a möbius strip of world and body in exchange [96]. This, in turn, has repercussions for the use of the health care system. Women who do not know how to describe pain as the health care culture describes it might not be perceived as being in pain. Chronic pain may be overlooked and other pains not used as a signal for illness. It also has repercussions for one’s sense of self. If the body is viewed as mutilated or strange, one is much less likely to present it for inspection as is necessary when seeking health care.

#### Taking a situated lens to the literature on health outcomes

Applying a situated, intersectional lens and taking into account SES alongside natal and diasporic health practices reveals that the research on FGC has typically failed to recognize differential outcomes of FGC. Within natal countries, SES determines whether a girl receives local anesthesia prior to the procedure, which in turn leads to less pain during the cutting itself (although it does not mitigate the pain subsequent to the anesthetic wearing off) [18]. Reviewing the literature from the perspective of place and outcomes reveals that where women live is critical even to reproductive health outcomes. For example, the outcome of delayed second stage labour and increased numbers of caesarian sections may be different in North America and Europe than in the natal countries [97]. Reports of delayed second stage labour come out of the natal country literature and not the North American/European literature. Caesarian sections are done because of difficulties in delivery in the natal countries while the high number of them in North America and Europe is due to physicians not being knowledgeable about how to do a vaginal delivery of a woman with infibulation [98]. Furthermore, in the diaspora, a cultural fear of caesarian section on the part of women with FGC seems to influence the use of prenatal care as well as a timely arrival at the hospital during contractions. This in turn leads to birth complications but they are not due to FGC, per se [99]. Adding to this are complications of race that may also intersect with health care provision in the diaspora while it may not directly in natal countries. Interestingly, there is scant literature interrogating racial bias as a mediator of obstetric outcomes for women with FGC in the diaspora.

As this example demonstrates, an intersectional approach can both extend biomedical explorations beyond a one body system and also inform a more accurate reading of the biomedical literature. Considering the intersections of traditional practices, biology, gender, and race led to new insights on the effects of a traditional practice. In particular, Somali-Canadian women in Toronto with Pharonic circumcision were found to have chronic pain that was unexpressed because of the cultural norm, ‘everybody has it so why talk about it?’ Moreover the very meaning of pain to the women was about incapacitation and not sensation. So asking about different types of pain as described in the West not only informed the researchers about the pain women had, but brought new realizations to the women themselves of their bodily sensation [18].

From a social justice perspective, intersectionality underscores the shortcomings of the biomedical treatment of women with FGC. For example (1) they have been treated as a single group of women independent of where they reside, their class, the circumstances of their FGC, and any other health issues besides reproductive health and sexuality, and (2) the main health concern is reproductive health [95]. However, for these women, many intersecting social locations shape not only their health but also the meaning of FGC, which in turn affects what is taken to be important about their health and their bodies. Ultimately, the entwinements of traditional practices, biology, gender, and race shape their views on both the normal body and pain, which in turn may well have negative effects on the quality of their health care interactions. Access to this more complex and sophisticated knowledge not only helps to empower the women themselves in terms of new/different interpretations of their experiences, but also provides different information for health care systems to improve approach/treatment, help overcome stigma, and remedy inequities.

### Example 4: CVD – race, gender, class, and other inequalities shape lay understandings of illness

As stated in the introduction to this paper, one of the ways intersectionality differs from the biomedical and even the ecosocial models is that it compels reflection on the social shaping of what types of evidence of health inequalities ‘count’ as credible. Shim’s [79,100] research exemplifies this call to examine whose knowledges matter. Through a content analysis of literature on the use of race and ethnicity, sex and gender, and social class in epidemiological research; observations of epidemiological and biomedical scientific conferences; and in-depth interviews with 21 cardiovascular epidemiologists and 24 people of color diagnosed with hypertension or coronary heart disease, Shim juxtaposed epidemiological and lay perspectives on the causes of heart disease. She explored whether, when, and how knowledge about CVD inequalities demonstrated an understanding of those inequalities as being intersectional (or not). This research takes seriously the commitment of an intersectional approach to value the lived experiences and situated, embodied knowledges of oppressed groups and individuals [101].

In doing so, Shim found that in contrast to epidemiologists who considered differences of race, ethnicity, SES, and sex as individual-level, demographic (and often biological) variables in isolation from one another, lay people living with heart disease attributed their heart disease to intersecting dynamics of race, gender, and class, as dimensions of social inequality. Lay people articulated nuanced understandings of the synergistic relationships between group status, relations of power, and well-being. Their accounts assert that the health effects of racial, class, and gender differences are mediated through profoundly and intrinsically social processes, that operate together to structure everyday experiences and life chances that in turn affect their risks for disease. In sum, lay people’s own accounts of disease causation counter reductionist practices of biomedical science. For example, one study participant, Mabel Rodriguez, a Mexican American woman with severe hypertension, described how hiring practices produced a racialized and gendered occupational hierarchy:

Oh, my days, people were very prejudiced! I mean they [white people] got the best jobs … It was all underneath the table … Higher up, a white person rather than the Latino or black. I knew what my place was there … A girl came in and was light-complected and … I would go in or somebody else darker than me would go in. They’d hire her. I’ve seen that happen. There was a lot of prejudice. It was most always swept underneath the table. Oh, we went through all that prejudice in our days. They used to call me ‘Mexican greaser’ … We got the dirty jobs while the others got the clean jobs. It’s always been that way.

If she had not been Mexican American, Ms. Rodriguez felt she would have had ‘different kinds of jobs and easier jobs, and a more calm life than [she] was having.’ Instead, she spent a lifetime of working long hours in low-skilled, low-wage, physically taxing jobs, which she felt deeply contributed to her hypertension. And, Ms. Rodriguez’s mostly solo parenting – an all-too-often gendered burden of reproductive labor and stratified reproduction – was, in her eyes, part and parcel of her experiences as a working-class Mexican American woman in the labor market. This all also took place within a wider institutional, infrastructural, and social service context that made it exceedingly difficult to access child care and maintain a household as a single working parent.

Ms. Rodriguez’s account is clearly one that highlights the effects of gender hierarchy on women’s bodily well-being. But these gendered dimensions of risk are always classed and raced as well: while the unequal burdens of reproductive labor tend to fall generally on women as a larger group, such labor is also stratified in racialized and classed ways, exacting a disproportionate toll on working-class women and women of color. When Ms. Rodriguez recalls lighter-skinned girls getting the job (or better jobs), she points to the effects of racism in constraining her access to the labor market – but at the same time, the very labor market for which she is deemed eligible is shaped not just by her race but also by her gender and class. Ms. Rodriguez, like Shim’s other participants, does not reduce race and gender to issues of class, or race and class to issues of gender. In these ways, lay people’s accounts of what makes them sick exemplify this paper’s central argument: that an intersectional framework can better account for heterogeneous and complex differences, and go beyond analyses that prioritize sex and gender.

As significantly, the lay narratives about disease coincide with the intersectional view of SDH, as being *synergistic with* but also *fundamental to* biological determinants of health. Biomedical and even sophisticated epidemiological accounts of disease typically seek to identify and isolate the most proximal factors associated with illness. Even the ecosocial model, by emphasizing how the social ‘gets into the body,’ can inadvertently focus attention on downstream and more proximate chains of disease causation. In contrast, the lay participants featured in Shim’s research constantly talk back and forth among political, social, economic, behavioral, and biological notions of etiology, reinforcing how the complex confluence of intersecting conditions, dynamics, and processes – including the biological – produces disease. In this way, their narratives insist on the conjoint importance of the biological along with the social, but also the causally fundamental importance of the social, by arguing that determinants of health both interact with and also underlie bodily processes.

This is the reality that people with heart disease live with, a reality that is situated and stratified. It is also a reality – or better, ‘slices of reality’[102,p.38] – that does not currently figure much, if at all, in research on cardiovascular risk. But the narratives also say something of the kinds of fundamental sorting and stratifying mechanisms that produce cardiovascular health inequities, and that should be explored as part of scientific research on heart disease. The causal accounts of heart disease by those afflicted strongly suggest that weaving the intersectional perspectives of subordinated groups into biomedical research will expand the evidence base [101] for public health, clinical, and social policies that can substantially alter existing patterns of morbidity and mortality.

Thus, true to intersectionality’s commitment to social justice, Shim’s participants lay out an agenda. First, in order to truly transform the distribution of health and illness – itself a key mandate of intersectionality approaches – we must intervene in the fundamental social, economic, and political processes, relations, and systems of power that produce health inequities. Second, we must also intervene in the practices, processes, and systems that shape the production of ‘official’ and legitimate knowledge about health. These interventions must include (but are not limited to): retooling and transforming conceptual models of disease incidence and distribution to account for complex intersections of disease determinants; diversifying definitions of expertise to incorporate lay and experiential knowledge; and rethinking scientific research priorities, criteria, and cultures to prompt and shape changes in scientific practices and beliefs about what constitutes science itself. Such changes are paramount to shift what we can know about disease and its distribution in ways that serve to open up – rather than constrain – the possibilities for health equity and social justice.

## Conclusion

As the examples in this paper illustrate, explicit integration of intersectionality into health research is not only possible but can make significant contributions to advancing work on biological/social entwinements. While engaging in such research, including working across disciplines, is challenging and moreover time consuming at each step of the research process, the knowledge generated warrants this new way of approaching health inequities work.

An intersectional lens shows that while health is experienced at the level of the individual, individual health outcomes and inequities, manifested in the body, are inextricably linked to interacting processes and structures of power at multiple levels. The examples demonstrate the new kinds of knowledge and evidence that can be produced when researchers take into account multiple levels of analysis bridging the biological, interpersonal, institutional, and societal. They show the importance of using multiple methods and privileging the experiences and perspectives of affected populations. And they advance understandings of why and how health is shaped so profoundly by time and place. If these dynamics were more systematically prioritized in research, different types of health problems, diseases, and illnesses would be more accurately understood and in turn, treatment opportunities, effective interventions, and necessary policy changes might be more clearly delineated and pursued.

In sum, synergies between biomedicine and social science advanced by intersectionality promise a more sophisticated, complex, and accurate understanding of health and its structural drivers. And because of the commitment to social justice enshrined in an intersectional framework, this form of entwinement also necessitates thinking beyond the research itself to what solutions, changes, and transformations are needed for the promotion of well-being among individuals, communities, and the wider society. Such outcomes and priorities are in line with ongoing global efforts to find innovative solutions to persistent and, in many cases, growing health inequities.

## Availability of data and material

Data included in this paper is available from the corresponding author on reasonable request.

## Paper context

This paper contributes to literature examining complex relationships between biological and social dimensions in the field of health inequalities by exploring how to combine biomedicine and intersectionality. It offers examples in HIV, post-traumatic stress disorder (PTSD), female genital circumcision/mutilation/cutting (FGC), and cardiovascular disease (CVD). The paper provides evidence to show how an intersectional approach can further research that integrates biological and social aspects of human health and generate more precise evidence for effective policies and practices aimed at tackling health inequities.
